# Ageing shift workers’ sleep and working‐hour characteristics after implementing ergonomic shift‐scheduling rules

**DOI:** 10.1111/jsr.13227

**Published:** 2020-11-09

**Authors:** Kati Karhula, Tarja Hakola, Aki Koskinen, Tea Lallukka, Anneli Ojajärvi, Sampsa Puttonen, Tuula Oksanen, Ossi Rahkonen, Annina Ropponen, Mikko Härmä

**Affiliations:** ^1^ Finnish Institute of Occupational Health Helsinki Finland; ^2^ Department of Public Health University of Helsinki Helsinki Finland; ^3^ Institute of Public Health and Clinical Nutrition University of Eastern Finland Kuopio Finland

**Keywords:** intervention, night shift work, pay roll data, social and healthcare, well‐being, working time

## Abstract

We studied whether implementing binding ergonomic shift‐scheduling rules change ageing (≥45 years) social and healthcare employees’ (mean age 52.5 years, 95% women) working‐hour characteristics (e.g. weekly working hours, number and length of night shifts, and short shift intervals) and sleep. We compared an intervention group (*n* = 253) to a control group (*n* = 1,234) by survey responses (baseline 2007/2008, follow‐up 2012) and objective working‐hour characteristics (intervention group *n* = 159, control group *n* = 379) from 91 days preceding the surveys. Changes in working‐hour characteristics were analysed with repeated measures general linear models. The fully adjusted model (sociodemographics and full‐/part‐time work) showed that proportion of short shift intervals (<11 hr, *p* = .033) and weekend work (*p* = .01) decreased more in the intervention than in the control group. Changes in sleep outcomes were analysed with generalised logit model to binomial and multinomial variables. The fully adjusted model (sociodemographics, full‐/part‐time work, job strain, health behaviours, and perceived health) revealed higher odds in the intervention group for long sleep (≥9 hr; odds ratio [OR] 5.53, 95% confidence interval [CI] 2.21–13.80), and lower odds of short sleep (<6 hr; OR 0.72, 95% CI 0.57–0.92), having at least two sleep difficulties often (OR 0.55, 95% CI 0.43–0.70), and more specifically difficulties in falling asleep (OR 0.56, 95% CI 0.41–0.77), waking up several times per night (OR 0.43, 95% CI 0.34–0.55), difficulties in staying asleep (OR 0.64, 95% CI 0.49–0.82), and non‐restorative sleep (OR 0.70, 95% CI 0.54–0.90) than the control group. In conclusion, implementation of ergonomic shift‐scheduling rules resulted in minor changes in ageing employees’ objective working hours and a consistent buffering effect against worsening of sleep.

## 1 INTRODUCTION

In western societies the work force is ageing. For example, the proportion of ageing (≥55 years) in the work force was one fifth in the European Union in 2018. The total proportion of all at least 55‐year‐olds is projected to increase from 33% in 2018 to nearly 41% in 2050 in the EU28 countries (European Commission, 2019). Even though the employment rate for those aged 55–64 years has steadily increased in the EU28 countries during the 21st century, their employment rate was still only 59% in 2019. The employment rate had an average of a 13% gender gap, being 53% for women and 66% for men (Eurostat, 2020). Moreover, ageing workers are also often employed in the social and healthcare sector, where providing services requires 24/7 shift work (24 hr/7 days of the week). Recently, a growing body of research has shown that shift work is associated with adverse health effects including increased risk of cardiovascular diseases (Torquati et al., 2018) and type II diabetes (Gao et al., 2020). However, different shift schedules are differently associated with adverse outcomes. Particularly night shift work is associated with severe negative health effects (Begtrup et al., 2019; Gu et al., 2015), whereas other shift characteristics, e.g. evening shifts, weekend work and short shift intervals are associated with insufficient sleep and recovery (Härmä et al., 2018; Wirtz et al., 2011).

Previous research unanimously supports fast forward‐rotating shift schedules (e.g. morning‐morning‐evening‐evening‐night‐night‐days off), as an ergonomic and health‐promoting option rather than more slow and/or backward‐rotating shift schedules (Härmä et al., 2006; Kecklund et al., 2008; Neil‐Sztramko et al., 2014). However, in healthcare, providing 24/7 services requires irregular shift work, and it is not exactly known how the more unpredictable and irregular shift schedules are associated with sleep. As a means to diminish the effects of irregular shift work, implementing some form of working‐time autonomy has been studied, but several of these studies have not found beneficial effects on sleep (Garde et al., 2011; Lowden & Åkerstedt, 2000; Nabe‐Nielsen et al., 2011). Due to individual preferences employees may prioritise longer continuous free time at the cost of optimising their sleep and recovery (Kecklund et al., 2008).

Ageing shift workers experience more sleep disturbances than younger ones (Hulsegge et al., 2019). Thus, when studying the effects of irregular shift work, employees’ age needs to be considered because the health risks seem to increase after the age of 45–50 years (Costa & Di Milia, 2008). Ageing is associated with difficulties in adjustment of circadian rhythms (Reinberg & Ashkenazi, 2008), increased sleep problems (Gander et al., 2019), and reduced tolerance for long working hours (Costa & Sartori, 2007). A previous study among older shift working nurses showed decreasing tolerance for shift work with increasing age, and poor scheduling practices were detrimental on their sleep and mental health (Clendon & Walker, 2013). Observational studies using self‐reported working‐time data suggest that older employees benefit from working hours that enable sufficient sleep and recovery at least as much as younger employees (Costa & Di Milia, 2008; Gander & Signal, 2008; Hakola et al., 2010; Viitasalo et al., 2015). For example, compared to slower backward‐rotation, older workers working in a quickly forward‐rotating three‐shift system had less sleep complaints than younger ones (Härmä et al., 2006; Viitasalo et al., 2015). In healthcare workers, improving working‐time ergonomics, mainly by reducing quick returns (short shift intervals of <11 hr) resulted in positive effects on heart rate variability (Järvelin‐Pasanen et al., 2013), sleep, alertness, and well‐being of all ages (Hakola et al., 2010).

Previous studies were mainly limited by using subjective data on working hours and only very few studies used objective working‐hour data when studying employees’ sleep. However, no previous study has investigated the effects on binding ergonomic shift‐scheduling rules in irregular shift work and their effects on sleep and actual working hours. Therefore, we investigated whether implementation of binding ergonomic shift‐scheduling rules changes ageing employees’ objective working‐hour characteristics and whether these rules have beneficial effects on ageing employees’ sleep duration and sleep difficulties.

## 2 METHODS

### 2.1 Study design and samples

This natural intervention study was based on two cohort studies, the Helsinki Health Study (HHS) and the Finnish Public Sector (FPS) study. The HHS has studied social and work‐related determinants of health since the year 2000, and the FPS study investigated working conditions, health, and well‐being utilising the Finnish 10‐towns study and Health and Well‐Being among Finnish Hospital Personnel Study, covering altogether ~30% of Finnish public sector employees. The participants were social and healthcare employees of the City of Helsinki (HHS, *n* = 253) in the intervention group and employees from the Finnish 10‐towns study (*n* = 1,234) in the control group. All the participants were: (a) employed in the social services and primary and specialised in‐patient care, (b) working in shifts, (c) aged ≥45 years at the baseline survey, and (d) born between 1945 and 1963. All the employees (both in the intervention and the control group) worked on a period‐based work contract (total planned working hours 114 hr 45 min in 3 weeks) and monthly salary. The intervention did not change the amount of working hours or grounds for payment in the intervention group. Overtime was avoided in both the intervention and control groups.

### 2.2 The ergonomic shift‐scheduling rules

The Social Services and Healthcare Division of the City of Helsinki has developed healthy and ergonomic shift‐scheduling since 2005. In primary healthcare in‐patient wards, ergonomic shift‐scheduling recommendations resulted in the first positive results in the “Healthy working hours” ‐ research and development project (Hakola et al., 2010; Järvelin‐Pasanen et al., 2013). As a continuum of implementing the ergonomic shift‐scheduling recommendations in primary in‐ and out‐patient care, specialised in‐patient care and elderly care, the Social Services and Healthcare Division put into operation binding ergonomic shift‐scheduling rules for the whole sector starting from 1 November 2011. Implementation was supported by coaching head nurses of the ergonomic rules in lectures and workshops, both in the social services and the healthcare division. The main alteration to shift schedules was a change from backward to forward rotation (Hakola et al., 2010; Järvelin‐Pasanen et al., 2013). Permanent night work was not allowed anymore. The ergonomic shift‐scheduling rules included the following concrete rules for the shift planner/head nurse:

1. The maximum number of total working hours in one 7‐day period is 50 hr.
2. The number of consecutive night shifts is 1–5.
3. Night shift is or nights shifts are followed by at least 2 days off.
4. The maximum length of night shift is 10 hr.
5. The number of quick returns from evening to morning shifts is reduced to a maximum of 1–2 per 3‐week roster.

The control group did not implement binding ergonomic shift‐scheduling rules. The scheduling fulfilled at least the conditions defined by the national legislation and collective agreements regarding, e.g. inter‐shift recovery times.

### 2.3 Participants

**Figure 1:**
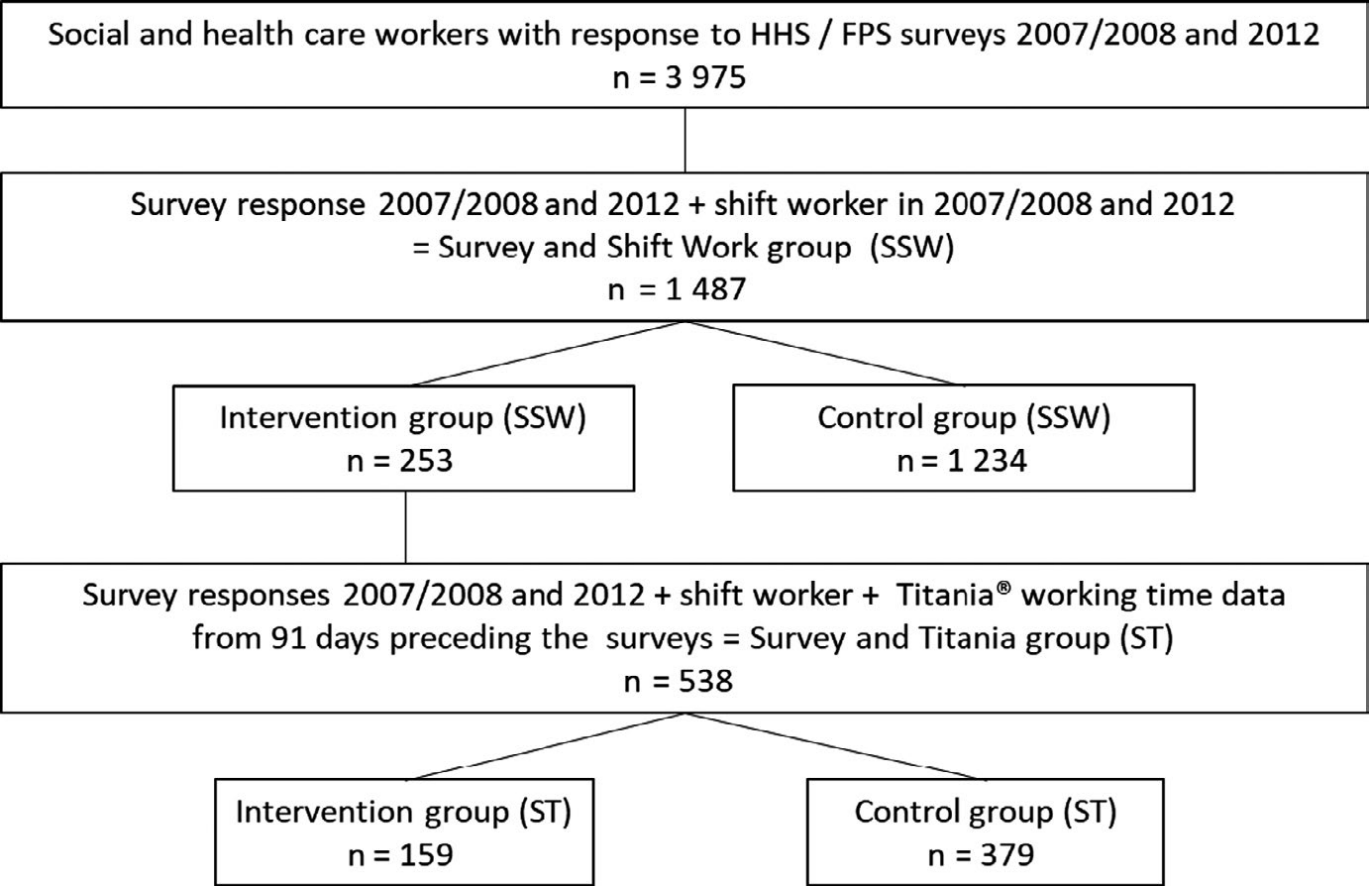
Flowchart of the study

The participants in the intervention group answered two consecutive HHS surveys, in 2007 (*n* = 7,330, response rate 83%) and 2012 (*n* = 6,802, response rate 78%); whereas the participants in the control group answered two consecutive FPS surveys, in 2008 (*n* = 14,053, response rate 72%) and 2012 (*n* = 13,883, response rate 71%; Figure 1).

In the survey and shift work group (SSW, *n* = 1,487) 95% of participants were women. Their mean (*SD*) age was 52.3 (4.55) years in the intervention group and 52.5 (3.93) years (*p* = .176) in the control group.

We additionally linked the 538 participants having objective working‐hour data from Titania^®^ shift‐scheduling software (CGI Finland Ltd) from 91 days preceding both of the surveys and having at least 31 work shifts during that time period, comprising the survey and Titania group (ST group). The methodology of retrieving and analysing the daily payroll working‐hour data has been described earlier (Härmä et al., 2015). In the ST group (*n* = 538), the sex distribution and mean age were similar in both groups (52.4 years, *p* = .918). According to the working‐hour data, 98% of the participants in the ST group were full‐time workers. The most common occupational groups in the ST intervention group were nursing assistants and other healthcare staff with shorter education (*n* = 46, 29%), head nurses and nurses (*n* = 39, 25%), and social workers and other social sector employees (*n* = 36, 23%); and in the ST control group were nursing assistant in in‐patient care (*n* = 143, 42% of those with job title available), nursing assistant in out‐patient care (*n* = 60, 17%), and nurse (*n* = 56, 16%).

The descriptive statistics of the SSW and ST groups are shown in Table 1.

**Table 1:** The descriptive statistics of the participants at baseline (intervention group 2007/control group 2008)

Variable	Survey data (SSW)			Survey + working‐time data (ST)		
	Intervention group (*n* = 253), % (*n*)	Control group (*n* = 1,234), % (*n*)	*p* ^a^	Intervention group (*n* = 159), % (*n*)	Control group (*n* = 379), % (*n*)	*p* ^a^
Sex						
Woman	88.5 (224)	96.7 (1,193)	<.001	90.6 (144)	96.6 (366)	<.009
Man	11.5 (29)	3.3 (41)		9.4 (15)	3.4 (13)	
Marital status						
Married/co‐habiting	60.3 (152)	69.6 (851)	.005	65.8 (104)	68.6 (260)	.544
Other	39.7 (100)	30.4 (371)		34.2 (54)	31.4 (119)	
Children aged <18 years^b^						
No	66.4 (158)	62.3 (548)	.257	62.1 (95)	55.6 (145)	.216
Yes	33.6 (80)	33.7 (331)		37.9 (58)	44.4 (116)	
Good perceived health						
No	23.4 (59)	30.8 (377)	.023	19.6 (31)	29.9 (112)	.018
Yes	76.6 (193)	69.2 (849)		80.4 (127)	70.1 (263)	
Current smoker						
No	69.4 (175)	65.0 (440)	.213	72.3 (115)	65.9 (145)	.217
Yes	30.6 (77)	35.0 (237)		27.7 (44)	34.1 (75)	
Alcohol risk use^c^						
No	83.7 (211)	88.7 (1,086)	.034	84.3 (134)	88.9 (337)	.153
Yes	16.3 (41)	11.3 (138)		15.7 (25)	11.1 (42)	
Physically active^d^						
No	27.1 (68)	27.0 (307)	1.000	26.4 (42)	26.1 (88)	1.000
Yes	72.9 (183)	73.0 (828)		73.6 (117)	73.9 (249)	

^a^ Fisher’s exact test.

^b^ Living in the same household.

^c^ Women ≥7 and men ≥14 alcohol portions/week (1 alcohol portion ~12 g of pure alcohol).

^d^ Exercise ≥3 hr/week.

### 2.4 The objective working‐hour data

The studied working‐hour characteristics from the employers’ registries included proportion of long (>40 hr) and very long (>48 hr) work weeks of all work weeks, proportion of long (>12 hr) work shifts of all work shifts, proportion of evening (starts after 12:00 hours and is not categorised as night shift) and night shifts (≥3 hr between 23:00–06:00 hours according to the Finnish Working Time Act), proportion of long (>10 hr) night shifts, proportion of ≥4 consecutive night shifts of all night shift periods, proportion of short recovery periods (<48 hr) after the last night shift of all recovery periods, proportion of quick returns (<11 hr) of all shift intervals <48 hr, proportion of single days off of all day off periods, and proportion of weekend work (Saturday and/or Sunday) of all weekends.

### 2.5 Survey data

As the main interest of this study, the following sleep outcomes were studied: average sleep duration was elicited with a question “How many hours do you normally sleep during 24 hr?” with multiple choices from ≤5 hr to ≥10 hr with 1‐hr intervals in the HHS survey (intervention group) and from <5 hr to >10 hr with 30‐min intervals in the FPS survey (control group). Sleep duration was categorised into three classes: “short” (≤6 hr), “normal” (FPS 6.5–8.5 hr, HHS alternatives 7 and 8 hr), and “long” (≥9 hr) sleep. Sleep difficulties (difficulties to fall asleep, waking up several times per night, difficulties in staying asleep, and feeling tired and worn out after waking up after usual amount of sleep (from now on ‘non‐restorative sleep‘ for brevity) during the last 4 weeks were asked with a scale from “not at all” to “every day” (Jenkins et al., 1988). The answers were dichotomised as having a sleep difficulty if the frequency was ≥2 times/week. All the sleep difficulties were analysed separately and additionally having at least two sleep difficulties often (≥2 times/week).

The following survey variables were used as covariates. Marital status was dichotomised as married or co‐habiting versus single, divorced, or widowed. Number of children aged <18 years living in the same household was dichotomised as having or not having children. Job strain was elicited with the Job Content Questionnaire (Karasek et al., 1998), with three items for job demands and nine items for job control (Lallukka et al., 2008). Job control and job demands were divided into “high” and “low” based on median values and then categorised into four: high control/low demands, high control/high demands, low control/high demands, and low control/low demands. Perceived health was measured using a 5‐point Likert‐type scale from good to poor (Blaxter, 1987) and was dichotomised as good perceived health with ″good” and poor perceived health with the two alternatives ″rather poor” and poor”. Physical activity was categorised as hours per week during leisure time and commuting in different grades of intensity (Haario et al., 2013). Those reporting >3 hr of physical activity per week were classified as “physically active”.

The survey question about cigarette smoking was dichotomised into current smoking and non‐smoking including also ex‐smokers. Drinking alcohol was elicited with questions on the consumption of bottles of ‘beer or cider’ and ‘wine or other mild beverages’ per week and ‘sprits’ as bottles per month. The total consumption was dichotomised with a threshold for alcohol risk at ≥7 portions/week (1 portion ~12 g of pure alcohol) for women and ≥14 portions/week for men.

### 2.6 Ethical issues

The Ethics Committee of the Department of Public Health at the University of Helsinki (30 November 1998) and the Ethics Committee of the health authorities at the City of Helsinki (5 October 1999) approved the HHS study and the Coordinating Ethics Committee of the Hospital District of Helsinki and Uusimaa (HUS) approved the Finnish Public Sector study (HUS 1210/2016). All the participating organisations gave written permission to use the employers’ working‐time registries for research. As working‐hour data are employer‐owned data, there was no need to gather individual employee’s permission for the data collection.

### 2.7 Statistical methods

The statistical analyses were conducted using IBM SPSS Statistics, version 25 (IBM Corp.) and SAS, version 9.4 (SAS Institute Inc.) software. Changes in the continuous objective working‐hour characteristics between baseline at 2007/2008 and follow‐up at 2012 were analysed with repeated measures general linear model (GLM). Maulchy’s test of sphericity showed no violation of sphericity and therefore no corrections were used. The within‐subject GLM models were run to calculate *F* and *p* values for both unadjusted and adjusted models. The fully adjusted model included age, sex, marital status, having children aged <18 years living in the same household, and full‐/part‐time work as covariates. Changes in the categorical sleep outcomes (sleep duration and sleep difficulties) between 2007/2008 and 2012 were analysed with a generalised logit model to binomial and multinomial variables. The fully adjusted model included age, sex, marital status, having children aged <18 years living in the same household, job strain, smoking, alcohol use, physical activity, and perceived health as covariates. In both the repeated measures GLMs and genaralised logit models, missing values in having children aged <18 years comprised an own class.

## 3 RESULTS

### 3.1 Changes in working‐hour characteristics

The objective working‐hour data (the ST group) showed that the proportion of short shift intervals and weekend work decreased in the intervention group, whereas short shift intervals decreased less and weekend work increased in the control group (fully adjusted model *p* values .03 and .01, respectively). However, the proportion of very long work weeks increased in the intervention group and decreased in the control group (fully adjusted model *p* = .02; Table 2.)

**Table 2:** Changes in average proportions (%) of the working‐hour characteristics between baseline (intervention group 2007; control group 2008) and follow‐up (2012)

Proportion of…	Intervention group (*n* = 158)		Control group (*n* = 373)		Unadjusted		Adjusted^a^		Adjusted^b^	
	2007	2012	2008	2012						
	Mean (*SD*)	Mean (*SD*)	Mean (*SD*)	Mean (*SD*)	*F*	*p*	*F*	*p*	*F*	*p*
long (>40 hr) work weeks of all work weeks	28.4 (15.5)	29.0 (18.3)	28.1 (15.6)	28.0 (17.9)	0.14	.71	0.12	.73	0.02	.88
very long (>48 hr) work weeks of all work weeks	4.2 (7.7)	5.5 (9.7)	5.6 (8.7)	4.0 (8.8)	6.80	<.01	7.67	.006	5.74	.02
long (≥12 hr) shifts of all shifts	1.7 (4.0)	1.3 (4.0)	2.4 (7.0)	1.6 (4.9)	0.59	.44	0.49	.49	0.47	.50
evening shifts of all shifts	28.1 (19.8)	25.4 (20.9)	28.5 (17.3)	28.0 (18.8)	2.59	.11	2.74	.10	3.45	.06
night shifts of all shifts	11.2 (26.6)	11.6 (26.5)	8.5 (19.5)	9.8 (22.4)	0.47	.49	0.60	.44	0.15	.70
long (≥10 hr) night shifts of all night shifts	7.2 (20.1)	6.5 (19.6)	4.5 (15.0)	5.4 (18.4)	1.29	.26	1.68	.20	1.68	.20
>4 consecutive night shift spells	17.4 (35.8)	18.4 (35.9)	10.2 (26.4)	12.6 (30.0)	0.35	.55	0.70	.41	0.61	.44
short recovery periods (<48 hr) of all recovery periods^c^	15.2 (31.4)	12.1 (23.5)	13.5 (25.3)	15.4 (24.5)	1.04	.31	0.42	.52	0.27	.61
short shift intervals (<11 hr) of all shift intervals^d^	16.3 (15.1)	11.7 (13.1)	19.5 (16.3)	17.6 (15.0)	3.99	<.05	3.35	.07	4.55	.03
weekend work of all weekends	36.0 (20.1)	34.1 (20.8)	37.3 (20.2)	40.0 (22.2)	7.01	<.01	8.00	<.01	6.75	.01
single days off of all days off^e^	21.8 (9.5)	23.2 (10.9)	23.5 (11.0)	25.7 (12.3)	0.19	.66	0.17	.68	0.12	.73

Results from repeated measures general linear model (GLM) presented as *F* and *p* values.

^a^ Adjusted for age and sex.

^b^ Adjusted for age, sex, marital status, children aged <18 years (missing value = own class) and full‐/part‐time work.

^c^ Intervention group *n* = 33, control group *n* = 89.

^d^ Shift intervals <48 hr included.

^e^ Intervention group *n* = 109, control group *n* = 282.

### 3.2 Changes in sleep length and sleep difficulties

At the baseline survey (SSW group, *n* = 1,487), lower proportion of the intervention group participants reported having often difficulties falling asleep and waking up several times a night than the control group participants (*p* values < .02). The intervention group also comprised a lower proportion of short sleepers (≤6 hr, *p* < .001). The groups did not differ regarding occurrence of difficulties in staying asleep and non‐restorative sleep (*p* ≥ .06). At the follow‐up survey, occurrences of all the studied sleep variables showed statistically significant difference between the intervention and the control groups. In both groups, a larger proportion of the employees reported short sleep length, difficulties falling asleep, and waking up several times a night than in the baseline. A smaller proportion in the intervention group reported difficulties in staying asleep and non‐restorative sleep than in the control group (Table 3).

**Table 3:** The proportions (%) and frequencies (*n*) of the sleep measures at baseline (intervention group 2007; control group 2008) and follow‐up (2012) surveys

	Threshold	Baseline					Follow‐up			
		All (*n* = 1,487), % (*n*)	Intervention group (*n* = 253), % (*n*)	Control group (*n* = 1,234), % (*n*)	*p*		All (*n* = 1,487), % (*n*)	Intervention group (*n* = 253), % (*n*)	Control group (*n* = 1,234), % (*n*)	*p*
Sleep length (hr)	≤6 hr	29.8 (438)	22.6 (56)	31.2 (382)	<.001^a^		35.3 (522)	29.2 (73)	36.5 (449)	<.001^a^
	6.5–8.5 hr^b^	69.3 (1,020)	74.2 (184)	68.3 (836)			63.3 (937)	66.4 (166)	62.6 (771)	
	≥9 hr	1.0 (14)	3.2 (8)	0.49 (6)			1.5 (22)	4.4 (11)	0.89 (11)	
Sleep difficulties^c^										
In falling asleep	≥2/week	17.2 (248)	11.3 (27)	18.4 (221)		.007^d^	19.6 (287)	12.2 (30)	21.1 (257)	.001^d^
Waking up several times/night	≥2/week	41.7 (601)	27.4 (67)	44.7 (534)		<.001^d^	47.5 (693)	29.1 (70)	51.2 (623)	<.001^d^
In staying asleep	≥2/week	28.1 (400)	23.4 (57)	29.1 (343)		.07^d^	33.3 (484)	23.0 (55)	35.4 (429)	<.001^d^
Non‐restorative sleep	≥2/week	30.2 (438)	25.1 (62)	31.3 (376)		.06^d^	33.5 (489)	24.4 (59)	35.4 (430)	.001^d^
Sleep difficulties (≥2) often	≥2/week	32.8 (484)	25.8 (65)	34.2 (419)		.002^d^	37.9 (560)	24.2 (61)	40.7 (499)	<.001^d^

^a^ Chi‐square test.

^b^ HHS survey alternatives 7 and 8 hr, FPS survey alternatives 6.5, 7, 7.5, 8 and 8.5 hr.

^c^ During the past 4 weeks, Jenkins Sleep Scale.

^d^ Fisher’s exact test.

Based on the changes in the sleep variables, the intervention group had a lower odds for short sleep length (≤6 hr; fully adjusted model odds ratio [OR] 0.69, 95% confidence interval [CI] 0.54–0.89) and higher odds of long sleep length (≥9 hr; fully adjusted model OR 5.73, 95% CI 2.28–14.40). The intervention group had a lower odds of having at least two of the studied sleep difficulties often, as well as a lower odds for each of the sleep difficulties separately (fully adjusted model ORs 0.43–0.67; Table 4).

**Table 4:** Changes in sleep outcomes between 2007/2008 and 2012 in the intervention group (control group as a reference)

		Unadjusted	Adjusted^a^	Adjusted^b^
		OR (95% CI)	OR (95% CI)	OR (95% CI)
Sleep length (hr)	≤6 versus 6–8.5 hr	0.71 (0.57–0.89)	0.72 (0.58–0.90)	0.72 (0.56–0.92)
	≥9 versus 6–8.5 hr	5.13 (2.64–9.98)	4.62 (2.34–9.15)	5.53 (2.21–13.8)
Sleep difficulties^c^				
In falling asleep	≥2 versus ≤1/week	0.54 (0.40–0.72)	0.55 (0.41–0.74)	0.53 (0.39–0.74)
Waking up several times/night	≥2 versus ≤1/week	0.43 (0.34–0.53)	0.44 (0.35–0.54)	0.42 (0.33–0.53)
In staying asleep	≥2 versus ≤1/week	0.63 (0.50–0.80)	0.64 (0.51–0.80)	0.62 (0.48–0.80)
Non‐restorative sleep	≥2 versus ≤1/week	0.66 (0.53–0.82)	0.66 (0.53–0.83)	0.67 (0.52–0.86)
Sleep difficulties (≥2) often^c^	≥2 versus ≤1/week	0.56 (0.45–0.69)	0.56 (0.45–0.70)	0.55 (0.43–0.70)

Results from generalised logit model to binomial and multinomial variables presented as odds ratios (ORs) and their 95% confidence intervals (CIs).

^a^ Adjusted for age and sex.

^b^ Fully adjusted model age, sex, marital status, children aged <18 years (missing value = own class), job strain, perceived health, smoking, alcohol risk use, physical activity.

^c^ During the past 4 weeks, Jenkins Sleep Scale.

## 4 DISCUSSION

The aim of the present study was to investigate how implementation of ergonomic shift‐scheduling rules changes ageing employees’ objective working‐hour characteristics and whether organisational level ergonomic rules effect ageing employees’ sleep length and sleep difficulties. Several parallel changes towards better shift ergonomics were observed in the working‐hour characteristics in both the intervention and the control groups. As the main result, the present study showed a larger decrease in the proportion of short shift intervals and weekend work in the intervention group than in the control group. The change in proportion of very long work weeks showed an opposite result. The intervention group had consistently longer sleep length and better sleep quality.

To the best of our knowledge, there are no earlier studies comparing the proportions of the studied working‐hour characteristics and implementation of ergonomic shift‐scheduling rules in irregular shift work. Previous studies have mostly investigated the effects of implementing fast forward‐rotating shift schedules to regular shift work (Härmä et al., 2006; Kecklund et al., 2008; Neil‐Sztramko et al., 2014) or increasing working‐time autonomy in irregular shift work (Garde et al., 2011, 2012). Therefore, comparisons to earlier research are few.

In the present study, the implementation of ergonomic rules resulted in a large decrease in the proportion of short shift intervals. This is a beneficial change, as there is accumulating evidence on the negative effects of short shift intervals (Karhula et al., 2017; Vedaa et al., 2016, 2017). Employees themselves also rate short shift intervals amongst the most problematic shift characteristics (Åkerstedt & Kecklund, 2017). According to the objective working‐hour data, the proportion of weekend work also decreased in the intervention group and slightly increased in the control group. However, we assume that this change might be more related to changes in organising assisting nursing work than to the working‐time ergonomics intervention. However, the change is beneficial for the employees’ well‐being, as especially Sunday work is associated with poorer work‐life balance and occupational accidents (Wirtz et al., 2011).

One unfavourable working‐hour characteristics change was found, as the proportion of very long work weeks slightly increased in the intervention group but decreased in the control group. However, the interpretation of this finding merits caution, as the overall proportion of very long work weeks was very low (~5%) and the observed change was very small (1 unit). It is highly questionable whether this change is relevant in practice. Overall, the present study found more effects on working‐hour characteristics than a study of implementation of participatory working‐time scheduling software (Karhula et al., 2020), which suggests that binding ergonomic rules should be used to achieve health‐promoting, ergonomic changes in working‐hour characteristics among ageing employees.

The present study showed consistent results regarding improved sleep, as the intervention group was less likely to have short sleep length and any of the studied sleep difficulties in comparison to the control group between baseline and follow‐up. The effects on sleep were partly due to more negative change in the control group compared to the intervention group, which, however, shows that ergonomic working‐time rules can have buffering effects towards worsening of sleep among ageing shift workers. A recent field study found similar results regarding short sleep duration and age; older shift workers had over seven‐times more often shorter sleep duration between night shifts compared with work‐free days, unlike younger shift workers (Hulsegge et al., 2019). Earlier intervention studies aiming to improve adaptation to shift work have mostly focussed on working‐time autonomy, and they often have not found an effect on sleep (Garde et al., 2011; Lowden & Åkerstedt, 2000; Nabe‐Nielsen et al., 2011), whereas studies implementing fast forward‐rotating shift schedules have mostly resulted in improved sleep estimations amongst other beneficial well‐being effects (Härmä et al., 2006; Viitasalo et al., 2015).

It seems that some shift schedule regularity resulting from regular forward‐rotating schedule or ergonomic rules that restrict strenuous shift combinations are needed to promote sufficient sleep amongst shift workers. Policy‐level actions are also shown to be effective. Consistent with the present study, Ropponen et al. (2017) found several positive changes in shift ergonomics, e.g. in recovery times and realisation of shift wishes after changes in collective agreement. All in all, an intervention aiming to improve shift ergonomics in irregular shift work can have multiple beneficial effects on employees’ well‐being via improved sleep. Previous HHS studies have shown an association between deviating sleep lengths and weight gain (Lyytikäinen et al., 2011), as well as an association between frequent insomnia symptoms and heavy drinking and physical inactivity (Haario et al., 2013). However, there was also a small, but significant group of long sleepers in the intervention group. Long sleeping has previously been associated with incomplete recovery (Härmä et al., 2019). Although the number of long sleepers was low both in this study and in Härmä et al., (2019) study, the studies indicate a need for more research among long sleepers working shifts.

The main strength of the present study is the use of a validated method to collect objective working‐hour data. This enabled us to study very detailed and accurate exposure on the studied working‐hour characteristics. We were able to investigate also working‐hour characteristics that were not included in the ergonomic shift‐scheduling rules, e.g. proportion of evening shifts and single days off, as changes in one working‐hour characteristics affect to other characteristics as well. Additionally, previous research is mostly based on self‐reported working‐hour data, which has been shown to be affected by recall bias (Härmä et al., 2017), especially when studying complex or irregular working‐hour characteristics, such as length of work shifts or shift intervals. Even though the implemented ergonomic shift‐scheduling rules can be seen as a continuum of several research and development projects, we were able to confirm using objective working‐hour data from the whole intervention organisation, that the largest change in, e.g. short‐shift intervals took place between the years 2010 and 2012, (Finnish Institute of Occupational Health, 2019) thus between the surveys used in the present study.

Some limitations need to be addressed. The present study had a limited sample size of employees having both objective working‐hour data in the baseline years and having answered the surveys. We were unable to investigate how the actual implementation processes were carried out, and it is possible that other actions in the intervention organisation have had a beneficial effect, e.g. on employees’ workload and consequently, to their perceived well‐being.

As the study used retrospective collection of the objective working‐hour data, the surveys had a 1‐year time difference at the baseline. However, the follow‐up surveys in 2012 in both studies were conducted with a few months difference and the HHS 2012 survey was conducted 11–12 months after implementation of the binding ergonomic shift‐scheduling rules in the intervention organisation. There was a difference in the most common occupational groups between the intervention and the control groups, as 23% of the participants were social workers in the intervention group versus very few in the control group. However, all the included employees were shift workers who also had night shifts, and the social workers included worked in round‐the‐clock services, e.g. in nursing homes for children, disabled persons, and elderly. Moreover, some of the measures were not optimal in their precision as, e.g. the Jenkins Sleep Scale includes the term night‐time sleep, which is not ideal when studying shift workers. The generalisability of the results to other working‐time arrangements may be limited, as the shift arrangements in the Finnish healthcare sector are somewhat irregular even after the implementation of the ergonomic rules.

The present results can be utilised in designing ergonomic shift‐scheduling principles for irregular shift work. The results support implementing binding ergonomic rules among ageing employees when aiming to achieve sufficient sleep quantity and quality with increasing age. Even though the actual significant changes were observed in few of the studied working‐hour characteristics, previous research confirms our understanding that these characteristics are amongst the most problematic to employees. However, the results would merit confirmation in a larger sample, preferably with different occupational sectors.

## 5 CONCLUSION

Among ageing social and healthcare employees, the implementation of ergonomic shift‐scheduling principles was followed with minor changes in objective working‐hour characteristics and a consistent buffering effect to worsening of sleep.

## CONFLICT OF INTEREST

The authors report no conflicts of interest.

## AUTHOR CONTRIBUTIONS

All authors (KK, TH, AK, TL, AO, SP, TO, OR, AR, MH) took part into designing of the study. Data manager AK was responsible for calculating the working‐time variables and general data management. Jaana Pentti (University of Helsinki) merged the HHS data with working‐time variables and pseudonymized the HHS data. KK and AO conducted the statistical analysis. KK drafted the first version of the manuscript. All authors contributed to the writing of the manuscript.
